# Mr. Travers on Dissection, or Poisoned Wounds (Second Article.)

**Published:** 1826-10-01

**Authors:** 


*An Inquiry concerning that Disturbed State of the Vital Functions, usually denominated Constitutional Irritation.* By Benjamin Travers, Esq. &c. &c.

### ON POISONED WOUNDS.

[Second Article.]

Besides the personal dangers to which the daily avocations of the medical man exposes him, from coming in contact with diseases of a contagious nature, there are some others to which he is subjected while cultivating his professional knowledge, purely for the sake of science. None of these are more terrible and sudden in their direful effects than wounds received while prosecuting the study of anatomy—or investigating the seats and nature of disease. By these, the student is sometimes rapidly hurried to the grave, in the hey-day of youth, and the hopes of fond parents thus closed for ever:—by these, the experienced practitioner is torn from his family, in the midst of health and rising reputation, while his widow and orphans are all at once precipitated from affluence to indigence! No event is more calculated to awaken the best sympathies of our nature, or call forth unavailing regret, than one of this description. The melancholy accident too often happens to the zealous and indefatigable student, while his idle, dissipated, and careless companion escapes. The conscientious and the anxious medical practitioner, who steals an hour from necessary repose, for the sake of ascertaining the nature of his patient's complaint, when art had failed, is thus suddenly caught in the fangs of death; while the incurious and routine practitioner, who seeks and thinks he needs no lights from morbid anatomy, safely plods on in his dull and darksome path.—Such are the events of life as daily displayed before us. They are calculated to call forth the sigh of sorrow—but we dare not repine at the ways of Providence, dark and mysterious as they sometimes seem!

The class of diseases, on which we are about to enter, embraces a wide range, and is not confined to the anatomist and pathologist. Veterinary students, farriers, butchers, cooks and others, in common with ourselves, are especially exposed to what are termed "poisoned wounds" and their fatal consequences. Our readers are aware that some writers and teachers have denied the absorption of putrid matter or morbid poison in these cases, attributing the whole of the phenomena to a certain predisposed irritable state of constitution, induced by impure air, sedentary habits, deficient rest, anxiety of mind, depressing passions, &c. &c. We have never denied that this predisposition in the constitution would often render the slightest puncture dangerous in its consequent inflammation; but we have, at the same time, maintained that this explanation frequently fails, and that we are forced to admit the introduction of a virus, on many occasions, to account for the train of dangerous symptoms succeeding slight dissection wounds in people of excellent constitutions, and in the prime of life and health. We are happy to find that Mr. Travers has not been led away from the sober path of observation by this fashionable doctrine; and that he recognizes two distinct classes of these diseases—one arising from simple injuries in irritable constitutions, of which numerous examples have been given in our first article—the other, from the operation of an inoculated poison.

"The following case demonstrates that inflammation is not necessary to the most virulent and fatal action of the poison, and in general, I should be disposed to say of these cases, that the symptoms of local inflammation and constitutional irritation exist in an inverse ratio of severity. The following case rests upon the authority of Sir Astley Cooper.

"Mr. Elcock, student of anatomy, slightly punctured his finger in opening the body of a hospital patient, recently dead, about twelve o'clock at noon, and in the evening of the same day (Monday) finding the wound painful, showed it to Mr. Cooper, after his surgical lecture, by whom he was referred to Dr. Haighton, in whose house Mr. E. at that time resided. He applied a poultice to the finger, and took some active aperient medicine. During the night the pain increased to extremity, and symptoms of high constitutional irritation presented themselves on the ensuing morning. No trace of inflammation, however, was apparent, beyond a slight redness of the spot at which the wound had been inflicted, which was a mere puncture. In the evening he was visited by Dr. Babington, in conjunction with Dr. Haighton and Mr. Cooper. Still no local change was to be discovered, but the nervous system was agitated in a most violent and alarming degree, the symptoms nearly resembling the universal excitation of hydrophobia; and in this state he expired at three o'clock on Wednesday morning, within the short period of forty hours from the injury." 204.

If the above case do not indicate the introduction of a morbific virus, on as good evidence as medicine can generally afford, then we are very blunt in our perceptions.

Mr. Travers classes inflammations of the limbs from injury, according to the four several textures which are subject to these phlogoses: viz.—inflammation of the absorbents and their glands, superficial or deep-seated—of the veins—of the cellular membrane—of the thecæ of tendons and fasciæ of muscles. But although these forms of inflammation are sufficiently distinct in their origin, they are liable to become complicated in their progress.

The inflammation of the absorbent vessels has long been known and understood. It is characterised at first by inconsiderable swelling, while the blush is confined to the raised and cord-like absorbent lines, as is the chief pain. Above the elbow and in the axilla, the glands become swollen and tender, while a heavy pulsatile pain shoots from the wounded finger or hand upwards along the arm. In a short time, the inflammation, if acute, extends to the fasciæ, and the whole limb becomes swelled, heavy, tense, and morbidly sensible to the least impression. The constitutional symptoms are agonizing pain—sympathetic fever of suppuration—and afterwards irritation from the confinement of matter. The pain, in the first stage, has often caused delirium, and has sometimes been followed by fatal collapse, when active measures were neglected.

Inflammation of the deep-seated absorbents is thus characterized.

"A very slight and transitory visible inflammation of the superficial absorbents following an injury to the finger, is sometimes accompanied by acute pain and tenderness to the lightest touch, of the inner side of the upper arm and axilla; shortly, a deep-seated and firm swelling, marked by a vivid blush, occupies the channel of the large vessels from a little above the elbow to the hollow of the axilla. The depending position of the limb, which is more tense than swollen, cannot be borne for an instant. The pain is attended by inflammatory fever, and only relieved by free blood-letting. The blood bears the stamp of acute inflammation. The disease terminates in humeral, or axillary, or pectoral abscess, properly so called. This is inflammation of the deep-seated absorbents, and the slight and transient blush in the course of the superficial vessels, if present, is sympathetic." 207.

*Phlebitis.* This inflammation Mr. Travers considers as rare, and generally arising from the employment of a foul lancet in venesection, or something unfavourable in the constitution. It is recognized by moderate swelling but great tension of the limb—cordy hardness of the vein, traceable towards the axilla—excruciating pain—sense of weight and immobility—festering or oozing of the orifice. Such are the local or external phenomena; while the extreme constitutional excitement, morbid vigilance, mental irritability and despondency, running rapidly from delirium to exhaustion, mark the internal workings of this dangerous disease. The blood drawn is strongly cupped and buffed—the pulse rapid and stringy, becoming fuller and softer after venesection, the pain being only partially relieved. This inflammation is slow in affecting the contiguous textures, and terminates in a free purulent discharge from the ulcerated orifice, with one or more abscesses in the course or vicinity of the vessels. If overcome, the disease leaves a state of extreme debility, with hardness and stiffness of the limb.

*Inflammation of Cellular Membrane.* This is divided by Mr. Travers into simple or phlegmonous—serous or erythematic—erysipelatous—and gangrenous. On each of these our talented author makes many judicious observations; but we can only dwell for a few minutes on the serous or erythematic phlogosis, which Mr. Travers considers as "a specific inflammation peculiar to poison." This species passes over or slightly affects the absorbents and the entire extremity—and shews itself in a slight fulness and tenderness of the neck, subclavian, humeral, and pectoral regions. The inflammation spreads backwards over the scapular region, and downwards by the serratus and latissimus dorsi. An erythematous blush, of a pink hue, irregular in its outline, but abruptly defined, next appears.

"If the finger is placed within the disc of color, it gives acute pain, so that the patient convulsively shrinks, while beyond this, pressure gives no pain. After a day or two, the part loses its vivid efflorescence, and becomes less exquisitely sensible; but the appearance of fulness rather increases, and the sensation to the touch is that of a very obscure or diffused fluctuation, as if the cellular membrane was broken up, a doughy or quaggy feel. If punctured, a serous fluid only escapes. If the patient survives a fortnight, this becomes purulent, in short it is a diffused cellular suppuration." 211.

In most instances the superficial absorbents are slightly inflamed for a day or two before the blush appears; but this is trifling and evanescent. Mr. T. considers this inflammation as produced by the specific irritation of a poison conveyed by the wound into the current of the circulation. It terminates in either a serous or lymphatic effusion into the cells, with an early disorganization of their texture—or, in its milder form, in a weakly and diffused suppuration throughout the cellular texture, unpreceded by any adhesive action. This disposition bounded for the most part to one side, appears afterwards in large undefined collections in different and distant regions of the body—and, if the patient live long enough, in very slow succession.

Mr. Travers touches on inflammation of the aponeuroses, thecæ, fasciæ, and periosteum, but his observations require no notice here. In recapitulation, he states it as his experience and belief, that the erythematous inflammation belongs to a specific irritation—the erysipelatous, to a specific state of constitution—the gangrenous, to age or constitution, if not plainly occasioned by the extent of mechanical disorganization.

All inflammations, he properly observes, are indications of natural resources. The erysipelatous and erythematous seem to be modes of inflammation, with inadequate power to carry them on to a termination. The adhesive stage is defective—they are incapable of healthy suppuration, and their imperfect effusion or suppuration is at the expense of life.

"Now this is either due to the nature of the injury, or to the state of the system. A poison taken into the system will set up this inflammation in a healthy subject; in an unhealthy subject such an inflammation will be produced by a simple injury; or a poison may be generated in the constitution, independent of local irritation. In scrofula the inflammation is always weak, and the secretion imperfect; in cancer this is yet more defective and vitiated, as is evinced by the deviations from a healthy character in the purity, consistence, color, and odor of the matter. Some poisons deprive the blood of the coagulating principle when drawn from the body. In such a condition of the system and the material as this change implies, is it possible that healthy secretions should be formed? Is it extraordinary that, in particular circumstances, the vascular action should be so modified or altered by an enervation of vital power as to be incapable of forming the first products of sanatory inflammation—incapable of circumscribing and determining secretions to the surface, and thus operating its own relief? Either these inflammations are constitutional, i. e. the result of certain unhealthy conditions of the system, whether arising spontaneously or from injury; or they are the result of specific irritations, the nature of which is to destroy the principles by which the constitution is preserved from destruction. Such in my belief is the explanation of the erythema and erysipelas set up by poison, whether extraneous or morbid." 222.

A bold inflammation of absorbents, cellular membrane, fascia, or even of a vein, is a much less dangerous action than that which manifests its character in the first instance by its effects on the constitution. When called to students or others labouring under such inflammations, he has regarded them in a favourable light, "for if they are to be referred to the inoculation of the wound with a poison, it is a local poison, its virulence appears and is exhausted upon the part—the constitutional disorder, however severe, being such as belongs to ordinary irritation." When, on the other hand, the degree of inflammation is disproportionate to the pain and nervous excitement—when the local affection shews itself, not upon the arm, but on the breast, and assumes the character of erythema or erysipelas, with which the constitution sympathises—then our author entertains a less cheering view of the case.

As an illustrative example of the symptoms marking the highest degree of constitutional irritation resulting from the poison of dead animal matter, our author introduces the case of the late Dr. Pett, of Hackney, as drawn up by Mr. Toulmin, Jun. of the same place. As the particulars of this interesting case are already before the public, we shall only merely glance at it here, to keep up the thread of the subject.

Dr. Pett examined the body of a lady who died of peritoneal inflammation, *post partum*, on a saturday morning. He handled the diseased parts, and assisted in sewing up the body. He was not conscious of having pricked himself. Between eight and nine o'clock in the evening he joined a party at a friend's house—the last he was ever to join!—and there began to feel heat and pain in the middle finger. On examining it by means of a lens and strong light he discovered a minute opening in the cuticle, in the centre of a blush of redness. This was touched with nitrate of silver, and afterwards with nitric acid. At 9 the next morning, Mr. Toulmin found the finger swelled and inflamed; the inflammation extending in red lines up the fore-arm. The countenance was now haggard and depressed—pulse 90—had had a shiver in the night, followed by some reaction, the pain in the finger being agonizing. He had taken ten grains of calomel in the night, and now took senna and salts—leeches—poultice. At 1 o'clock, the alteration in Dr. Pett's countenance was striking—the face was rather suffused—eyes hollow and ferretty—breathing sudden and irregular—his manner torpid, as if he had taken opium. Said he was completely knocked down, and had no strength left. The finger and hand were now more swollen, the skin of the former exhibiting a livid appearance, with subjacent effusion. A lancet was pushed in to the bone—the last and middle phalanges were found to be completely gangrenous. The inflamed absorbents could be traced to the elbow. The remainder of this day was chiefly passed in heavy sleep, with much suffering in the intervals. On Monday all things were making progress towards a fatal termination. Sir Astley Cooper thought he felt obscure fluctuation in the hand, and made an opening, through which issued a minute quantity of matter. Tuesday, all the symptoms aggravated. The powers of life were ebbing fast. On Wednesday morning there was an appearance of tranquillity and cheerfulness, but with some wandering of the intellect. His general appearance was frightful and haggard. He died at 6 o'clock on this day.

*Dissection.* The heart was of extraordinary size—the parietes thin, and substance flabby—no disease of valves or great vessels.* "The lungs were choaked with air," (an expression we cannot well understand) and contained much blood—texture sound. There was nothing else worthy of notice in the viscera. The head was not examined.

Mr. Travers next introduces several cases, among which we find that of Mr. Dease, the full details of which we gave in a former Number, from the third volume of the Dublin Hospital Reports. These cases we perfectly agree with Mr. Travers in considering as well-marked instances of the admission of a poison into the system. It is impossible, we think, for any unprejudiced man to read the cases of Dr. Pett, Mr. Dease, Mr. Delph, and others, without being fully convinced that a morbid virus was introduced into the system, whatever susceptibility there might be in some of the individuals to give that virus an increased facility of developing its fatal ravages throughout the constitution.

"All these cases exhibit strong excitement in the commencement, and early and rapid prostration. In none is any marked or permanent affection of the absorbent vessels apparent during life, yet the cellular substance of the axilla and breast are in all affected with erythematous (serous or suppurative) inflammation. In all the brain and nervous system sympathize quickly and deeply; in some the stomach and diaphragm are affected, and not in others. In one case the local inflammatory disposition is stronger than in another. It is by no means to be supposed that the causes which act in aggravation of common inflammation are suspended in cases of poison, when inflammation is once set up. There may be therefore more or less of acuteness and strength in the local action, according to the habit of the subject and the circumstances of the injury, as the nervous excitement may be more or less rapid and considerable in different individuals, under similar degrees of intensity of the poison. In drawing the line, therefore, between the simple and the specific action, we must bear in mind that to a certain extent they necessarily co-exist, and this adds considerably to the difficulty of the distinction." 287.

Mr. Travers next introduces a case communicated by Dr. Spurgin, illustrative of the minor degrees of irritation, local and constitutional, to which cooks and others, engaged in handling putrid animal matters are exposed, when they happen

* Dr. Pett considered himself affected with disease of the heart, and the above condition of the organ proves that he was right in his suspicion.

to have chapped or scratched fingers. The inflammation attending these accidents is generally of the erythematous species; appears in patches; is sometimes accompanied by intense throbbing pain; and, though subject to shift its position, does not quit the vicinity of the wound. It seldom advances to suppuration; but the health and spirits are materially affected. We shall present our readers with a sketch of Dr. Spurgin's case.

*Case.* A cook, of unhealthy appearance, was practising the art of boning on a stale hare the 4th December. In this operation she received two slight scratches, to which she paid no attention at the time. A few days afterwards they began to inflame, attended with dull pain and sense of numbness. A poultice was applied, and some aperient medicine taken. These relieved her for a couple of days, when the symptoms returned, and Dr. Spurgin ordered some colocynth and hyosciamus to be taken, and a poultice to be applied. Next day (11 Dec.) bark and soda was ordered, but this combination failed to allay the constitutional irritation—and on the 12th, she was attacked with violent pain in one of the fingers, and strong pulsation. An incision was made at her own request, and a sanious blood was discharged. Instantaneous relief followed. 13th. Free from pain—but no appetite—tongue charged and rather dry—dejection of spirits—weak unsteady pulse. 14th. The pain returned—the inflamed parts were dark-coloured, and the inflammation extending. Five leeches were applied, and produced relief. 15th. Another return of the pain and inflammation, requiring leeches, fomentations, &c. 16th. Nearly the same, and requiring similar treatment—constitutional symptoms increased. She was now sent into the country, and proper injunctions given her, where she soon recovered.

These kinds of cases, Mr. Travers thinks, have the specific character, and "the circumscribed sphere of the morbid action may be due to the very superficial injury inflicted, and, consequently, the imperfect exposure of an absorbing surface—or to the mere application of the matter to such a surface in place of its introduction by inoculation;—from either of which causes it might result, that the quantity of matter absorbed was so minute, or its virulence so modified, as to expend itself upon the parts contiguous to the wound, thus operating as a strictly local poison." We confess that our ideas do not entirely coincide with those of our talented author on this point. When we consider what dreadful consequences result from the slightest abrasion sustained during the examination of a body dead of peritoneal inflammation, we cannot but suspect that it is to a difference in the quality of the morbid poison, rather than to any difference in the quantity or mode of its application, or the extent of surface to which it is applied.

The next class of cases on which Mr. Travers enters are of more frequent occurrence and of less severity than the foregoing. "Although presenting very diverse phenomena, they are, I believe, generally, but not universally, considered to owe their origin to a similar cause with the foregoing. It is of importance to examine and decide this question." The following case, communicated by Dr. Gordon, of Finsbury Square, is introduced, among others, as illustrative of this class.

*Case.* Mr. Clifton, of Islington, aged 37, scratched his thumb with a needle, while sewing up the body of a female who had died of peritoneal inflammation, not puerperal; the scratch being too slight to attract attention. He was awakened at three o'clock the next morning, however, (12th Sept.) by the excruciating pain of his thumb extending along the arm to the axilla. Dr. G. saw him at 1 o'clock, P.M. and found the thumb and fore-arm swollen, with hardness of the absorbents, but no redness. The hand and arm communicated a burning sensation to the touch, the rest of the body being below par in temperature. The constitutional symptoms were very striking—the appearance of the patient brought to Dr. Gordon's recollection the symptoms of hydrophobia. Though naturally of firm nerve, the patient's whole appearance was that of extraordinary anxiety. He was morbidly sensible to the least noise, and was disturbed even by the breathing of surrounding persons. The countenance was shrunk, haggard, and cadaverous—head-ache and sensibility to light—intolerable sense of oppression on the chest—tongue loaded—nausea. He occasionally sprang out of bed, apparently unconscious of what he was doing, but really from pain and a resolution to avoid complaint. The pulse being very hard and full, at 120 in the minute, and his constitution being naturally good, he was bled to 30 ounces, having taken a dose of calomel in the morning, by which his bowels were well cleared. He was relieved by the bleeding, and the pulse fell to 80, though without much diminution of volume or hardness. Blood not buffy, nor firm in crassamentum. 13th. Had passed a bad night, and suffered dreadfully from pain; yet the constitutional symptoms were milder. The arm and hand were much swollen, and red lines were perceptible in the course of the absorbents. "He appeared much sunk in every respect, and complained greatly of his head." Leeches and cold applications to the head. 14th. Appearance improved, but he suffered dreadfully from pain in the night. The local affection was on the increase. Venes. ad ℥xij.—leeches in great number to the arm. By these he was relieved for a time, but an exacerbation occurred in the evening, with "the worst constitutional symptoms." Complaining of overwhelming oppression in the head and chest, the patient was again bled to sixteen ounces, leeches and purgatives, being reiterated. By these means, especially the bleeding, he was much relieved, both locally and constitutionally. 15th. Still in a very precarious state, all the symptoms being aggravated, except the affection of the head—pulse 120, and wiry—no appearance of matter in the hand—no rigor. An incision was made into the thumb, but only blood issued—it was then laid open to the bone, along its whole extent, and about a tea-spoonful of matter flowed from the point originally punctured. The matter was healthy—the relief instantaneous—sound sleep followed. In a few hours hæmorrhage, to the amount of 30 ounces, took place from the hand. After the faintness occasioned by this loss, the pulse rose to 120, full and hard—and the deep-seated pain in the arm returned. Leeches to the number of 140 were applied between Sunday and Tuesday. These alone afforded relief.

"' On the evening of Tuesday, the 16th, a sudden alteration took place. I was with him at the moment, and nothing could be more striking. The countenance became cold and cadaverous, and he threw himself back on his bed, abandoning the sitting posture, which was the only one he had hitherto been able to bear. He sighed frequently, tossed his arms about the bed, and rolled from side to side. The pulse rose to 140, became soft and compressible, but there was no diminution of pain. The symptoms appearing to me now more those of irritation than inflammation, I gave him twenty-five drops of Battley's solution of opium, and subsequently fifteen more. I may here observe that he had not been able to take opium in any form, and even hyosciamus disagreed violently with him, for on the second day, his pulse having been reduced by the bleeding, and suffering much from pain, I gave him five grains, which instantly affected his head and increased his restlessness. In the present instance, given just at the moment its exhibition was indicated, it was attended with the happiest effect, and he enjoyed some hours of refreshing sleep." 301.'

The application of a few more leeches was the only thing necessary. He gradually improved in health and strength.

Mr. Travers here records some other cases of a similar kind, namely, the cases of Mr. Brayne, now surgeon of Banbury—of Mr. Wansbrough, of Fulham—of Mr. Percival, a medical student, and others. We do not consider it necessary to quote any besides the one detailed. Mr. Travers looks upon these cases—Mr. Clifton's for example—as "cases of simple irritation." "The disturbance of the nervous system, it is true, was unusually great, but the rapidity and obscurity of the suppuration, the unretrievable confinement of the matter, and the aggravation of acute inflammation from this cause, sufficiently explain the overwhelming pain, and thence the whole train of symptoms." This is a plausible explanation, and probably a just one—but we confess that it is not quite satisfactory to us. We are aware, indeed, that severe constitutional disturbance will often result from matter confined under fasciæ or periostea; but a train of phemonena resembling hydrophobia the very day after the infliction of a dissection-wound, appear to us out of all proportion to those which would accrue from the most active inflammation and rapid formation of matter. It is allowed that a morbid poison was applied to the wound, and occasioned the local phenomena. Now we see no reason why the absorption of a portion of this said poison might not have *contributed* at least to the production of those terrible constitutional symptoms so rapidly developed after the infliction of the wound. This, however, is matter of conjecture. The agonizing pain arising from the inoculation of this destructive virus, whatever it is, may be capable of exciting extraordinary commotion in the nervous system, and, through that, of the whole animal economy.

"The case of Mr. Hutchinson related, with that of Mr. Dease, by Dr. Colles, in the second volume of the Dublin Hospital Reports, I consider an unequivocal example of absorption. The milky vesicle upon the site of the wound, the intense pain in the shoulder, the extensive erythema on the right side of the trunk, together with the extreme dejection of spirits, and constitutional suffering, are truly diagnostic. Mr. Egan's case, described in the same paper, I reject—not because I doubt that much variety may exist in the seat and character of the local action, but because neither the constitutional nor local symptoms were characteristic; they were in fact such as a simple local irritant was both likely and competent to produce, viz. erysipelatous redness of the wounded thumb, pain passing up the arm, an enlarged gland at the edge of the pectoral muscle, with the deep-seated abscess of the axilla, and symptomatic fever of suppuration." 326.

Mr. Travers makes some observations on Dr. Duncan's paper in the first volume of the Edinburgh Medico-Chirurgical Transactions, a paper well known to our readers, from the extensive analysis we gave of it. He entirely coincides with Dr. D. in considering "diffuse cellular inflammation" as "the most frequent form of that severe affection which occurs from the application of the fluids of a dead human body to a wounded or abraded surface." The cases of Messrs. Blyth, Young, and Hersey, appear to Mr. T. to have been well-marked instances of absorption.—Most of the other cases recorded by Dr. D. he classes with that of Mr. Clifton, &c. ranking them as cases of irritation only, and unaccompanied by absorption. As our author attaches great importance, and for good reasons, to this distinction, we shall be pardoned for introducing a pretty long extract in this place, in order that Mr. Travers may explain himself in his own words.

"But it may be further asked—since we admit that the poison absorbed from dead animal bodies varies in degree—may it not also vary, like the morbid poisons, in kind, and hence the difference in phenomena attending upon wounds in dissection be explained? For example, in some cases only, are pustules, pimples, or vesicles found in one or more parts. In some the absorbent glandular system is chiefly affected, in others the cellular; in some the wound inflames, and inflammation spreads along the limb from the wound; in others the wound can scarcely be said to inflame, but the inflammation is acute at the opposite extremity of the limb, or courses over the trunk of the body; in some the primary inflammation is altogether insignificant, the pain and general disorder of the system enduring and most severe, and after a continuance of many weeks of extreme debility and emaciation a deep chronic abscess presents, and proves critical of the malady. Lastly, in other cases the inflammation subsiding favorably in the injured extremity, appears within a few hours in that of the opposite side, and terminates in deep and extensive suppuration, or gangrenous erysipelas. It would appear on the above hypothesis, that one form of the poison irritated upon contact and provoked resistance to its admission, that is, inflammation in all the parts through which it passed; and that another, by its silent and uninterrupted entrance, and instant admixture with the mass of blood, exhibited on the contrary its characteristic effects at other and remote parts of the system.

"The following objection to admitting the cases in question to be instances of absorption appears to me to be decisive; they differ in no important respect from simple injuries with clean instruments; from abrasion and bruise, or inflammation of the cutis even without læsion. From such causes inflamed absorbents and their glands, and continuous cellular inflammation and suppuration are constantly arising, and there is no evidence to prove that continuous inflammation ever arises from the passage of poisons into the mass of blood. On the other hand, distant, diffuse and superficial cellular inflammation is certainly not an effect of simple injury, but this is the prevailing characteristic of the local action in the most urgent and fatal cases. Now although it is in the highest degree probable that the poison varying in intensity should occasion a more or less severe disease, as we see in the cases related, it is as improbable that the character of the specific action, should be subject to variety: in a word, it is improbable that the effects of the poison, quasi poison, should be at one time such as we have described, and at another such as are liable to be produced and frequently are produced by simple causes of irritation.

"Are the cases then of non-absorption to be regarded simply as results of mechanical injury, and would the subjects of them have been liable to be affected by similar consequences from similar injuries inflicted with clean instruments, in pure air and wholesome occupations?

"I think I have drawn a line of distinction broad enough to be easily recognised, even by a superficial observer, between the cases of absorption and non-absorption. If therefore the consequences of the wounds from dissection are more serious than such as result from ordinary clean wounds, and which I am disposed to believe they often are, the difference must be attributed to some peculiarity attending them, local or constitutional. It is not necessary that a local irritant, acting chemically, should be received into the mass of blood to produce extraordinary local excitement. The matter of gonorrhœa is commonly a more severe local irritant than the matter of syphilis; and various substances employed in surgery, which furnish no evidence of their absorption, exemplify the same fact. The constitutional excitement is in these cases the legitimate consequence of the inflammation produced by the local irritant, and is either slight or severe according to the seat, nature, and degree of such inflammation. The chemical, therefore, are to be regarded as operating on the same principle upon the constitution as the mechanical irritants, where their tendency is to produce high local inflammation; and their action being not dissimilar it is probable that they act in concert, and that the irritation of an acrimonious substance, superadded to that of a penetrating wound, may determine the extraordinary activity and extent of the local inflammation, and by consequence of the constitutional disorder. *I conclude that it is therefore probable that the irritation in some of these cases, though strictly local, is in its nature such as to act upon the constitution with greater severity than that of similar clean wounds.*" 338.

The last sentence of this extract, which we have taken the liberty to mark in Italics, narrows the question considerably. If the irritation of dissection wounds, "though strictly local," has something in its nature to affect the constitution more than the irritation of clean cuts, we think it is wandering out of our way to look for that something beyond the introduction of a poison which, in numerous other instances, is admitted to be the grand agent of the constitutional disturbance.

Many persons, Mr. Travers observes, are disposed to refer the severity of the inflammation attendant on dissection wounds to peculiar irritability of constitution—instancing the number of students who cut themselves every season, and yet escape with impunity. There can be no question that idiosyncrasy—bad health—the depressing passions—the unusual and unhealthy occupation of dissection—the London air—and probably irregularity and occasional intemperance, must predispose to the reception of a virus, as well as render its effects more dangerous when it is received. This is no more than we see in many other cases where people of good constitutions, cheerful dispositions, &c. escape contagious diseases, while others, under different circumstances, fall ready victims to the prevailing malady. This view of the case, however, does not materially bear on the question of absorption or simple irritation. The predisposition of the individual will facilitate the reception of the poison, and also aggravate the effects of local irritation.

"I consider the constitutional state in the cases of absorption to be as much more severe than that which prevails in a large proportion of the cases of simple irritation, as the local is less so. The latter present the fever symptomatic of local irritation, or the fever of suppuration, acute or chronic. It takes its form and pressure from the local inflammation. If it be more violent in the first, or more protracted and exhausting in the second stage, it is nevertheless such in character as we are accustomed to see in local inflammations which have a different origin, and we treat it on the same principle. The former, if I judge rightly, is a constitutional before it is, openly at least, a local disease. It may even destroy without exhibiting, if we except pain, a local sign; the local sign may be either scarcely developed, or, on the contrary, may have approached a favorable termination, when the system exhibits symptoms of dissolution. There are, however, some cases of simple irritation, of not very rare occurrence, viz. the cases of true paronychia, or acute suppuration within the sheath of the flexor tendon, in which the general and high excitement of the nervous system is so sudden and peculiar,* that we cannot be surprised where other circumstances favor the conclusion, to find such instances recorded as cases of a poison absorbed. And as respects the constitutional phenomena, they might merit to be so regarded; for there is little, if any difference to be recognized between them in the acuteness of the suffering, the high nervous excitement, and the suddenness of the collapse.†"

In respect to cases of absorption of a poison, it may be asked why the first evidence of its introduction should be furnished at a distance from the part injured, while the part itself escapes with impunity? Mr. Travers admits the difficulty of the question, but attempts the solution of it. He observes that the fact of the intermediate part escaping, in acute inflammation and abscess of glands in which the absorbents of an irritated part terminate, is every day seen in the sympathetic bubo attendant upon gonorrhœa and sores on the penis, mammæ, fingers, &c.

"The virulent and irritating quality of a poison not formed by a specific inflammation and secretion of the part, as the morbid poisons, but introduced, ready made, as I might say, into the system, as that of decomposed animal matter, discovers itself by the same signs, but greatly augmented in rapidity and intensity, as regards constitutional excitement and pain, and with this remarkable difference—that the seat and extent of the pain and inflammation are invariably diffused instead of the contrary.‡" 342.

"* Case of Mr. Clifton, p. 294, and of Mrs. C. p. 370."

"† The more invariable and severe attack of rigor in the cases of absorption deserves to be noticed. In those of simple origin it is often wanting."

"‡ The tendency to diffused erythematous inflammation from the absorption of animal poisons is exhibited in the stings and bites of insects, and the effects of certain animal substances taken into the stomach."

The arrest of the animal poison by the axillary glands, in the first stage of its progress, determines the original focus of the inflammation and pain—these glands become congested, and partially obstruct the transmission of the poison into the circulation. The cellular tissue surrounding the glands inflames, becomes swelled with serous effusion, and the inflammation and effusion spread to the axilla and chest. The pain affecting the top of the shoulder may, Mr. T. thinks, be explained by the distribution of the muscular twigs of nerves supplied by the axillary plexus.

"If this theory be correct, the invariable locality of the primary inflammation, as well as the mere and transient irritation (if any) of the absorbents, is explained. If the poison so insidiously entered the system and mingled with the mass of blood as to excite no primary irritation, the natural sign of resistance to its admission, it would be difficult to assign a reason why its effects should have this or indeed any local manifestation. It is well known that a ligature on the limb will prevent the effect of the viper's poison on the system, and that violent erysipelatous inflammation ensues, as in the case of Mr. Butler, related by Dr. Duncan. The lymphatic glands act as a natural but unfortunately ineffectual barrier to the passage of the poison; effects not very dissimilar to those of the mechanical impediment, are produced in their immediate vicinity, and are most conspicuously apparent in parts where the connecting membrane is disposed in greatest abundance and laxity of texture. But although the irritant has this local manifestation, its passage is impeded only, not prevented, and the constitution, therefore, frequently exhibits disturbance sooner than the part, which in the instance related, p. 203, proved destructive, even before any visible sign of inflammation appeared." 344.

The occasional determination of inflammation on the opposite side of the trunk, or on the opposite extremity, is not entirely peculiar to cases of specific irritation—nor common in these. It is altogether inexplicable on any theory which has yet been suggested. The insulated patches of erratic erysipelas—the abscesses which occur at the subsidence of fever—nay, the local determinations of inflammation in general, are equally unaccountable phenomena.

In the cases in which the admission of a poison is undisputed, two or three obvious injuries arise.

"Is the frequent fatality of these injuries to be ascribed to the condition of remaining animalization which has hitherto resisted the operation of external agents? or to such an alteration in the quality of the animal fluids by the incipient process of decomposition as merely divests them of the properties of living matter? or to a state of putrefaction so advanced, as to be actively disengaging new and deleterious compounds?" 345.

We agree with Mr. Travers, that our present state of knowledge does not enable us to give any satisfactory answers to these queries:—and it is better to confess our ignorance than to assume knowledge which we do not possess.—It is a fact, however, that the larger number of cases of this kind which occur, have their origin from dissection of bodies dead of inflammation, by which an abundant secretion of morbid fluids is accumulated, and amidst which the thoracic and abdominal viscera are handled by the operator. But a historical survey of the cases on record shews us that no condition of the dead subject is exclusively prejudicial, since a fair proportion of the sinister accidents had their origin in the dissection of stale bodies—namely, bodies that had been buried.

"If, as is probable, at the moment of expiring vital influence, or of de-animalization, new combinations give birth to a specific matter of contagion, it is to be presumed that the ultimate state of dissolution and decay which we term putrefaction, so alters its quality, as to neutralize and render it inert. And it is in conformity with observation that actual putrescency is in some degree a security from the effects of this species of injury. Putrid matters act as a strictly local poison; they irritate and inflame the part wounded and its immediate vicinity, and the constitution is in proportion protected." 347.

The changes which take place in vegetable bodies at the expiration of life, and prior to their ultimate decay, seem to support the foregoing opinions or rather conjectures; but as yet we know little of the matter. The progress of chemical knowledge will probably one day throw some light on the subject. In the mean time, the pathologist will do well to pay strict attention to the habit of the individual affected—to the nature of the wound—the fatal disease, whether acute or chronic, of the subject whence the poison was received—and the varying state of decomposition in the interval which has elapsed between death and dissection.

Another enquiry into the consideration of which our author enters is, the possibility or probability of a specific contagion being communicable after death, as that of erysipelas, small-pox, or syphilis. Although our author believes that erysipelas is sometimes contagious in the living subject, he thinks the question cannot be decided as to its *post-mortem* transmission by any facts yet in our possession. It is probable, however, he remarks, and scarcely doubtful, "that a pustular or vesicular disease, like small-pox or cow-pox, retains, for some time after death, the property of communicating the local poison, by reason of the favourable circumstances in which, for a given time, the virus is preserved." Students dissecting small-pox subjects have had variolous pustules of a spurious description form upon their wounded fingers. This is common; but Mr. Travers never knew an instance in which the constitutional disease was communicated. The effectiveness of the living poison preserved for many months by exclusion of the atmosphere, is obviously a distinct fact, not bearing upon the present question.

A specific morbid secretion, as that of syphilis, is capable of transmitting the constitutional disease from one living system to another. Thus, our author has known an accoucheur with a sore on his finger become affected with ulcer, eruptions, nodes, &c. bearing the character of syphilis, and which were only cured by mercury, in consequence of delivering a female affected with a venereal ulcer in the vagina. But our author has never known an instance of such disease being communicated by wounds received in the dissection of subjects dead of lues or cancer.

"Veterinary surgeons receive the poisons of glanders both from the living and dead animal by inoculating chaps and punctures, for the matter secreted in the cellular abscesses of persons so affected is capable of producing glanders, when introduced into the system of the horse or ass by inoculation. This is sufficient to shew that the specific property of the poison is retained in its passage through the human system, but there is no evidence of its acting on the human body otherwise than as the poison of dead animal bodies. The matter absorbed from the living animal affected indeed in a remarkable instance, which I shall presently detail, the glands at the angle of the jaw, and the mucous membrane of the nares, which are the proper seat of glanders in the horse and ass. This was, however, in common with a similar affection of the same textures in other parts of the body." 351.

*Case.* Mr. Turner, a veterinary student, injured his finger in examining the head of a horse which had died of glanders. An ulcer followed, with inflammation of the absorbents and cellular membrane of the hand, and symptomatic fever. In a few days an abscess formed on the opposite arm, and another on the lower part of the back. Matter taken from the abscess of the arm was sent to Mr. Coleman, who inoculated an ass with it, and produced fatal glanders. Mr. Turner's health was seriously affected—he became hectic—and was sent to Brighton, where abscesses formed in the lungs, kidneys, and other parts of the body, which ultimately wore him out. Matter was taken from an abscess in one of his knees, with which another ass was inoculated, and died in eleven days glandered. The following extract of a letter to Mr. Travers from Mr. Coleman, we think deserving of a place in our journal.

"You know that the seat of acute glanders in horses and asses is the secreting membrane of the nostrils, and that in farcy the superficial not the deeper seated absorbents are affected. The matter of both diseases in quality is the same. We can procure glanders from farcy matter, and farcy from glandered matter; and yet it appears that in the human subject, although susceptible of irritation from glanders and farcy matter, and capable of secreting a large quantity of the same poison, the seat and form of the disease are generally dissimilar. As far as my experience goes, the nostrils of the human subject are not susceptible of glandered ulceration or inflammation. In Mr. Turner's case, the superficial absorbents and cellular membrane were the only parts affected; but in other instances I have seen the superficial absorbents go on to suppuration and ulceration between the valves of the absorbents, in consequence of local irritation and the contact of farcy and glandered matter, corresponding in a great degree to the same disease in horses. From these facts, and many medical students falling a sacrifice to irritation from wounds in dissection, I have been led to believe that in such cases absorption of some poison frequently takes place. I am aware of the fact, that simple wounds with clean instruments sometimes occasion irritation of the absorbents, but I apprehend it will be found that butchers and other persons in the habit of cutting animal substances in a fresh state, are by no means equally liable to constitutional or even local irritation in the same degree as medical students. I have further to add, that I think it highly probable, that in all contagious constitutional diseases, such as small-pox, measles, lues, cow-pock, hooping cough, and perhaps from absorption of putrid matter, and other animal poisons, the whole of the blood is contaminated. In acute glanders the blood is undoubtedly affected. I have produced the disease, by first removing the healthy blood from an ass until the animal was nearly exhausted, and then transfusing from a glandered horse blood from the carotid artery into the jugular vein of the ass. The glanders in the ass was rapid in its progress and violent in degree; and from this animal by inoculation, I afterwards produced both farcy and glanders. The facts mentioned by Mr. John Hunter to shew that the blood is not infected can be readily explained. The blood, although it may possess materials to irritate certain parts, may not irritate all parts. Calomel may inflame the salivary glands, and increase their secretion, but produces no increase of the semen or synovia. Turpentine may stimulate the kidney to secrete more urine, but has no effect on other glands, although absorbed and circulating with the blood. Most parts of every animal have probably some peculiar susceptibility, and non-susceptibility; so that the same cause will produce different effects." 356.

Mr. Travers introduces two cases in which the poison was absorbed from the living animal. The first was that of Mr. Nallen, a veterinary surgeon, of Kidderminster, who, while administering a ball to a glandered horse, had matter from his nostrils inoculated into an abraded spot on his thumb. In a few days the part became painful, the inflammation having an unhealthy appearance. The sore was followed by many others of a similar description, affecting the hand, glands of the axilla, nates, and neighbourhood of the knee-joint. The irritation of these sores deteriorated the general health, occasioning loss of appetite, &c. The blue pill, warm bath, and decoction of sarsaparilla, at length restored Mr. N. to health.

The other case was that of a stout hackney-coachman, who had a chap on his thumb infected by matter from the nostril of his horse. In six hours afterwards, he was seized with violent pain and swelling of the thumb—and on the third day he became affected with cold chills and giddiness, together with total loss of power in his limbs for several hours. The arm was streaked with red lines and excessively swelled. On the fourth day he was taken to Guy's Hospital, where he continued ill for 24 weeks. Superficial collections of matter formed successively in the course of the absorbents, with sloughing of the integuments, leaving extensive ulcers that discharged a fetid and unhealthy matter. The glands under the jaw and in the groin swelled, and he was affected with pain between the eyes and down the nose, and exulcerations of the membrana narium. He had much constitutional illness—loss of appetite—severe pains and swimming of the head—pain in the spine—thick urine—unhealthy motions, &c. Despairing of life, he quitted the hospital, and spent the remainder of the year at home, in a state of the greatest emaciation. He now applied to an old woman, who gave him a decoction of herbs, which he invariably vomited up, but to which he, nevertheless, attributed his ultimate cure, which gradually took place at the expiration of a twelvemonth from the receipt of the infection. Mr. Travers was informed that an ass, inoculated with the matter of this man's sores by Mr. Sewell, died glandered.

In reviewing these cases, and comparing them with those in which the matter was received from the dead animal, we cannot but perceive several points of analogy between them and those cases where the matter was received from the human body in dissection. It is Mr. Coleman's opinion that a poison is absorbed in the human dissection, the result of diseased actions in the living subject. To this opinion Mr. Travers objects, for several reasons, which have already been glanced at. Mr. Travers concludes that the poison is generated after death, and pertains to the process of decomposition in its early stage. At the same time he thinks it highly probable that the irritating qualities and intensity of the poison, and the rapidity of its development may be influenced by the morbid condition of the body at the moment of death.

In the cases of disease produced by contact with the flesh or blood of diseased animals, as those cases of the butchers of the Hotel des Invalides and others, related in the Memoirs of the Academy of Sciences, the symptoms, Mr. T. observes, are altogether different from those following dissection wounds, viz. erysipelas gangrenosum, enormous emphysematous swellings, livid pustules, and tumours ending rapidly in gangrene and death. Mr. Travers can discover no analogy between these and the consequences of dissection wounds; and the same may be observed of those various affections described by different writers in Europe and the West Indies, as following the eating of the flesh of animals that have died of contagious murrain.

Here Mr. Travers introduces an interesting letter from Dr. Farre, who, while practising at Barbadoes, had an opportunity of observing the effects of that singular disease, the "pestis Bovilla," which produces in the human body a malignant carbuncle. This is also produced by the contact of the fluids of the diseased animal, and even by the rudest form of dissection.

Another enquiry of importance is this :—Is there any thing in the character of the constitutional disorder which distinguishes the case of specific from that of simple irritation? Mr. Travers thinks there is not. The disease, he observes, has been mistaken for pneumonia, erysipelas, and acute rheumatism. "The fact is, that the constitutional disorder arises from the operation of the poison as an irritant on the nervous system, and not from the inflammatory action which we see. This is but a symptom, not invariably present, and always comparatively insufficient to account for the constitutional excitement. But inflammation is capable of acting as an irritant of the nervous system, nearly, if not quite as powerful as the poison; and the irregularity and vehemence of the constitutional disorder are occasionally as great in the cases of non-absorption." The following case is a recent example.

*Case.* Mrs. C. aged 40, of healthy but irritable constitution, while suckling, complained of pain in the knuckle of her middle finger, with slight febrile indisposition. This went off by a dose of salts and some lotion; but two days afterwards it returned, and extended up the arm, affecting the side of the neck. A slight wound was now discovered in the finger, but she knew not how it was occasioned. In a day or two more she became maniacally delirious, and a fulness and tension were perceptible over the flexor tendon of the finger. Her pulse was very quick, contracted, and a little irregular. There was neither absorbent, glandular, nor cutaneous inflammation, but a little fulness from the elbow upwards, and about the subclavian region. Mr. Travers laid open the theca of the flexor tendons from the second joint to the metacarpus; when a tea-spoonful of healthy pus issued. Twenty ounces of blood were drawn from the arm—the hand fomented—and calomel and salts exhibited. Next day the delirium was unabated. More cathartics—another venesection—hyosciamus and opium at bed-time. Some inflamed absorbents on the fore-arm—wound in the finger discharging copiously. She sometimes appeared a little better, and at others worse, but in the course of eight or nine days she sunk exhausted. An examination was made, and a long account given of it, exhibiting the diseased appearances in the arm, but these we need not detail. The viscera were not allowed to be examined.

It is a curious circumstance attending this case, that the maid-servant who fomented the lady's hand after the incision made into it, became affected with pain, inflammation, and swelling of one of her fingers, on which there had been no wound or scratch. These symptoms were accompanied by fever, and the inflammation spread to the lower extremity, producing an affection resembling phlegmatia dolens. She was cured by active depletion, and early and free opening of the abscesses.

The laundress, too, who had been employed to wash Mrs. C.'s sheets, had no sooner opened and immersed them in water, than she was overpowered by an effluvium of a peculiarly offensive nature, and instantly complained of a most severe pain darting into the axilla and shoulder. Nausea and faintness followed, and in the evening a rigor. Next day fever and delirium. No swelling or inflammation could be perceived in the painful parts; but two days afterwards, a deep pectoral abscess presented itself, which was opened, and discharged several ounces of healthy pus. She recovered after a tedious confinement.

Mr. Travers is disposed to think that these two latter cases were not mere coincidences.

"For example, the incessant steeping of the hand in hot fomentations was sufficient to render the skin preternaturally irritable, and the frequent contact in this state with the matter of a sore, might have irritated sufficiently for the production of common whitloe, a disease in many cases of seemingly spontaneous origin. The rapid diffusion of the inflammation over the fascia of the hand is accounted for by the circumstance first mentioned. The laundress's case appears to have been fever excited by the effluvium of morbid secretions. The cause and locality of the pain and of the abscess, which proved critical of the febrile action, I am entirely unable to explain. In neither this nor the former case was there any breach of the integument by which absorption could be facilitated. The laundress was attacked at one and the same instant with nausea and faintness from the stench, and with acute lancinating pain, from the handling of the linen; a woman who was present told me that she turned as pale as death, and resting her extended hand against the wall, exclaimed in agony, 'Oh God! my arm,' within two minutes of unfolding the sheets. This was surely the operation of a subtle poison on the nervous system. It could only be through this medium that it could operate so instantaneously. Much anxiety of mind prevailed and might have predisposed her to be severely affected, as she considered her livelihood involved in the issue of Mrs. C.'s illness." 382.

In respect to the lady herself, Mr. Travers asks, if she had absorbed a poison, how was it received? She had not been engaged in any culinary operations—the wound was a mere scratch—and she was quite unconscious how she had received it—most probably from a pin. "The difference," says Mr. T. "in severity of constitutional irritation from matter forming under the common fascia, as in what I call fascial abscess, and forming within the proper sheath of a tendon, is not unfrequently the difference of life and death. The former case is the common whitlow—this is the paronychia gravis. The occurrence of the former is as thirty, at least, to one of the latter. *There is no degree of constitutional excitement, however vehement, to which the confinement of matter within the proper sheath of a tendon is not adequate.*" If this last sentence be correct, we have, of course, a key to those terrible constitutional orgasms that take place sometimes where no suspicion of an absorbed poison existed.

In a former part of the volume, Mr. Travers has introduced the interesting case of Mr. Delph, surgeon, of Edmonton, as an unequivocal one of absorbed virus, similar to that of Mr. Clifton, which we have already detailed. In this place, Mr. T. gives us a second attack, which Mr. Delph experienced in the summer of 1824, but which Mr. Travers views as a good specimen of the characteristic difference between the simple and specific injury—to the former of which it belongs. We shall glance at this case, on account of the decisive treatment which was so successfully employed in it.

*Case.* While examining the body (on the 6th May) of a child that had died of croup, after measles, Mr. Delph punctured his left thumb with the point of the needle—the instrument having penetrated to the bone. Immediate acute pain was felt, and plenty of ablutions were employed. In the evening the pain became lancinating, and next morning the thumb was much swelled and red. Numerous leeches, and then poultices, were applied. By Mr. Travers' advice, a dose of calomel, followed by the black draught, was taken. On the second morning, the absorbents were affected to the axilla, and the whole arm was tense and painful. More leeches were applied. Mr. D. had one or two slight rigors, and he felt a good deal depressed. Mr. Travers now prescribed venesection to syncope, which required the abstraction of 35 ounces. "From that moment I became, by comparison, easy—a decided alteration was manifest in the appearance of the arm, the pain ceased, except in the thumb, which remained swelled and painful for several days, but by the constant application of poultices, suppurated in the punctured part, and healed kindly in about a fortnight."

"'In my former unfortunate accident you will remember, that the scratch in my finger was not noticed until it became inflamed, about sixteen hours after the accident, when I experienced some shooting pain in the part. At forty hours after the accident I had one of the most tremendous shivering fits I ever witnessed, which was succeeded by others, at regular intervals of fifteen minutes. These were followed by delirium and other symptoms of alarming constitutional excitement. Though the arm and hand became swollen, there was no blush to be seen on the integument, nor any indication of inflammation of the absorbent vessels; the integuments of the same side of the trunk, from the spine to the linea alba, took on the same swollen appearance with the arm; this was followed by erysipelas and diffused abscesses, which continued to form over different parts of the body for a period of three years, and at times rendered my existence miserable.'" 390.

We sincerely hope this zealous and intelligent surgeon will never again be visited by such a terrible tax on his pathological researches!

Mr. Travers observes that the occasional approximation of the constitutional symptoms, in the two classes of cases, "naturally leads to the belief that there is nothing in the nature of the malady which puts it beyond the pale of medical treatment in one case more than in the other."

"The difference amounts to this, that the poison is in one case the irritant, in the other the inflammation. In either case the local action may constitute so much of the disease as to demand vigilant treatment; but in the former, the disorder arises from the admission of the poison into the circulation, and in most instances the local action is insignificant, except as it demonstrates the existence and activity of the poison. To take up the local disease in this case as especially important, is to begin at the wrong end. The terms 'poison' and 'specific' have been somehow supposed to convey a mysterious import of incurableness, and we are apt to conclude that our labour is vain, and to yield the contest in despair, when conclusive evidence of absorption is at hand. Nothing can be more contrary to reason than this impression. If a common pathological character, viz. that of over-excitement of the nervous system, be established, the constitutional treatment of the cases will be similar. The difference in rapidity of development, and vehemence of action, must be met by a corresponding activity of treatment. If the pain anticipate other signs of inflammation, if the excitement be so intense and the depression of the nervous system follow so close upon the stage of excitement, as either to disguise the former or abridge it of its ordinary limit; we must not suffer ourselves to be betrayed into a hypothetical belief that the operation of the poison contra-indicates the employment of those measures, which have been found efficacious in proportion as they have been early resorted to, in cases to which these bear a strong and obvious analogy." 392.

Again Mr. Travers says:—"There is nothing in the nature of the irritation induced by specific agents, as the saliva of the poisonous snake or of the rabid dog, which warrants a belief that it resists the operation of remedies, provided the activity of the poison be not such as cuts off opportunity by a sudden arrest of the functions. It stands, in fact, on the same ground as irritation from other and simple causes, as from mechanical injury." We confess that this doctrine does not coincide with the ideas which we have formed of the action of specific poisons. The constitutional irritation arising from a severe whitlow, and that resulting from the bite of a mad dog, have very different *causes;* and as effects are modified by their causes, so we believe that these two irritations are very different, in kind as well as in degree—and, consequently, that the medical treatment must be different. It is true, the constitutional symptoms in poisoned wounds and in common injuries, often bear a great resemblance to each other; but our senses are very bad means of seizing those nice features of distinction which divide diseases of the most different or opposite characters. But, after all, experience is a better guide than theory upon these occasions; and we fear that the success which has hitherto attended the treatment of poisoned wounds will not greatly strengthen Mr. Travers' doctrine of their identity (as to constitutional effects) with other injuries.

Mr. Travers observes that if, from the moment of the shock, reaction fail, the injury is in its nature fatal, from the complete paralysis of the nervous system; but where this is not the case, we have no reason to conclude that the disease is incurable. Certainly not. But the question still presents itself, is the reaction to be met with the same remedies and to the same extent, as reaction from simple injuries? In the one case there is a poison circulating in the system, which Nature is endeavouring to repel—in the other, the cause is local, which makes, we apprehend, a considerable difference in the event. We agree with Mr. Travers that—"the effect of very gentle alteratives (as they might be termed) of the cerebral circulation, to preserve life when trembling in the balance, in the extreme states of excitement and depression, is continually seen in practice." It is upon such occasions indeed that the judgment of the practitioner becomes particularly conspicuous. The following case may serve as an instance, and it shews the necessity of treating symptoms as they rise, rather than blindly acting on any preconceived or predetermined system.

*Case.* "I was called a few weeks ago to an infant of nine months, the subject of very extensive burn three days before, whose state of exhaustion was extreme, and considered to be as it appeared, hopeless. The infant was without a pulse at the wrist, cold, sunk, and wan. I directed a tea-spoonful of brandy with three of gruel to be given every half hour until it warmed. This was strictly followed,—the infant revived, and the attendants, encouraged by the manifest improvement, persevered beyond the point prescribed. It became flushed, and when I again visited it the next day, had fallen into the state of apoplectic coma with sonorous breathing, having a fully dilated and motionless pupil. I immediately gave two grains of calomel, with directions to repeat the dose in four hours if it had not operated, and applied a leech to each temple, which bled freely. On the succeeding morning, I had the satisfaction to find the infant in the best possible state, with a steady circulation and lively expression, and from this time it went on without any relapse through the healing process, to complete recovery." 394.

### RECAPITULATION.

From a revision of the preceding cases and observations, it appears to our author that affections of the sensorium, such as acute bodily pain or mental anxiety, co-operating with local injury—a burn or a bruise—a laceration or a fracture—an operation or an inflamation—a hæmorrhage or a wasting suppuration—a poison permeating the body—are all so many mischiefs severally competent to produce states of the system which have a very distinct relation to fever, yet over which the remedies usually employed in fever have no control. That some phenomena are common both to fever and irritation is unquestionable—but the combination in fever presents a result totally dissimilar.

"When fever is symptomatic of local inflammation, its character corresponds to the boldness and activity of the inflammation, as in pleurisy or peritonitis, or to its insidiousness of commencement and slowness of development, as in scrofula. The preponderant influence of the nervous system, which characterizes irritation, appears to derange the sympathy and consent, the revolution and duration of actions constituting the paroxysms and types of fever, and referred to the struggles of a vis medicatrix. This fact is exemplified in the state denominated febrile irritation, which is symptomatic of minor injuries, and in the irregular train of symptoms which accompany disorganizing inflammation. In all fevers the nervous system is more or less involved, but for the most part secondarily or as a consequence, if we except the case of phrenitis; whereas in irritation from local injuries of a severe description, this system is first, and so affected, as apparently to prevent the formation of fever, and to present a series of symptoms sui generis." 401.

The slightest cognizable forms of constitutional irritation, observes our author, are among those trivial but common ailments which, characterized by languor and uneasiness rather than real indisposition, are usually regarded as hypochondriacal. He believes that these affections originate in a morbid sympathy with some local and temporary irritation. A proof of this local irritation is often obvious, as in indigestible food or some vitiated secretion in the first passages. It is truly astonishing what effects will be produced upon the whole nervous system, and upon the brightest of the mental faculties by an irritation of the nerves of the stomach, the duodenum, or the intestines. Individuals of strong susceptibility, who are unfortunately subject to these internal irritations, are branded with the reproach of hypochondriacism, and are told that they have only to exert the energies of their own minds to drive away the horrible and desponding ideas that harrass their imaginations. But no effort of the mind (per se) can effect this. The only relief is by improving the condition of the body. Remove the cause, which is always corporeal, and the effects will cease; but not till then. The reciprocal influence, however, of mind and body, is by no means well understood yet. A triste moral impression will quickly derange the functions of digestion and secretion; and the presence of the undigested aliment and of the vitiated secretions in the primæ viæ will repay with interest the injury which the body has received from the mind. A state of intellectual feeling is then produced which no language can describe, and which is ten thousand times worse to bear than corporeal pain. The miseries of human life are all exaggerated and cloathed in the most terrific colours—while the pleasures of existence can only be contemplated with horror or disgust. Such will often be the condition of an individual one moment, and in the next, after a copious biliary excretion—an imperious excitation—or some sudden and vivid mental impression, he will be completely metamorphosed into a being of an opposite character, with all his intellectual and physical powers in their pristine vigour!

Some hilly walks—brisk exercise—
Fling but a stone, the giant dies!

We shall, ere long, take up this interesting subject more fully, and hope to be able to throw some light upon many of its abstruse points. But to return.

Mr. Travers observes that we may freely admit, and without any reproach to our art, the utter hopelessness of that state of direct and extreme prostration which sometimes supervenes upon injuries, not in their own nature mortal. Still it is proper and necessary to study the phenomena of diseases over which we have, at present, little or no control—for it is impossible to predetermine the line beyond which a right understanding of theory, and a consequent right application of principles may not enable us to push our triumphs. Mr. Travers proceeds to recapitulate the leading points of the evidence presented to the reader in the preceding chapters.

"In the fatal cases of 'Burn' it will be seen that the state of prostration was consentaneous with the infliction of the injury; the period of survival varying from eight to fifty-eight hours, and inflammation had no share in the results.

"In the fatal cases of complicated injury, under the heads 'Fractures,' &c. and 'Operations for recent injuries,' there is some variety as regards the accession of the symptoms of prostration. Four days is the extreme period to which life was prolonged. The results in these cases were also independent of inflammation.

"The fatal cases of lithotomy, mentioned under the head 'Operations for chronic diseases,' were too rapid for inflammation to have any share in the results. Indeed none of the symptoms proper to the operation were present.

"The third section, which treats of 'Inflammation ensuing upon injuries and operations,' presents these symptoms setting in at a variable but considerably later period, as was to be expected, the inflammation standing in the relation of exciting cause.

"In the fourth section on 'Hemorrhage and colliquative Suppuration' the symptoms of prostration take a measure strictly according to the exciting cause—more rapid if from hemorrhage or its consequences, erysipelas or gangrene; slower if from wasting suppuration.

"Under the head 'Poison' I have included cases which, from their origin and circumstances, bear an ambiguous character, and which have been generally classed with those distinctly referable to this source of irritation. My motive in doing so was more forcibly to illustrate by comparison and contrast the points of distinction between the genuine and the spurious cases. In those, as I consider, cases of absorption, which terminated fatally, the appearance of the symptoms was alike early and the career rapid;* whilst those which recovered strongly resembled each other in the protracted disorder, local and constitutional, which supervened.† On the other hand in those cases in which the

"* Dr. Pett's, Mr. Dease's, Mr. Newby's, &c."
"† Messrs. Delph's and Smartt's."

symptoms are referred to the character of the injury and not to the poison absorbed, all the variety which might be expected is observable, in the accession, extent, and duration of the symptoms, according to the situation and severity of the local mischief.* These therefore properly belong in my arrangement to the third section: viz. Inflammation ensuing upon injuries and operations.

"I should thus epitomize the symptoms indicating the two forms of prostration:—

"1. Prostration without re-action is marked by universal pallor and contraction of surface, shuddering, very small and rapid pulse, astoundment of the mental faculties, generally a dilated pupil, shortened respiration, dryness of the tongue and fauces; indistinctness, and at length cessation of the pulse at the wrist, stupor, oppressed and noisy respiration, coldness of the feet and hands, involuntary twitchings, relaxation of the sphincters, confirmed insensibility, stertor, and death.

"2. Prostration with excitement is marked by the signs of languor and stupor or drowsiness in the commencement,† to which, after a variable interval, succeed rigor, precordial anxiety, restlessness, jactitation; a rapid and bounding pulse, oppressed respiration with frequent attempts to sigh, flushed countenance, contracted pupil, dry heat of skin, parching thirst, rejection of liquids taken into the stomach, incoherence and wildness of expression, sometimes amounting to fierce delirium. This state is succeeded by exhaustion marked by somnolency, a profuse chilly and clammy sweat, a haggard and livid aspect, a small irregular or fluttering pulse, innumerably rapid, panting respiration, passive convulsions, hiccup, and subsultus, the stupor and stertor of apoplexy, and death." 407.

The above are outlines of the general character of the two forms. There are some varieties. A variety of the first form consists in little more than a state of continued languor and faintness, with coldness and sleepiness verging on deliquium, the pulse and breathing being scarcely perceptible, and terminated by convulsions. A variety of the second form consists of an alternation of coma and convulsive paroxysms, in which the features undergo the contortions and fixedness of epilepsy, vehement maniacal ravings, and impotent attempts to rise from the bed, with an incessant muttering, terminating in exhaustion.

These are extreme states. There are others in which the symptoms of prostration gradually give place to a partial and

"* Messrs. Clifton's, Brayne's, Slight's, &c."

"† These, like other of the symptoms enumerated, are of course subject to exception. Hours have sometimes passed away without the occurrence of an alarming symptom, in which case a severe rigor, or several at short intervals, generally open the attack; in other instances this symptom is wanting."

defective reaction, protracting life, but faintly improving the prospect of restoration, which remains doubtful for several days in succession. Or, on the other hand, an efficient and healthy reaction may be quickly set up, consequent upon symptoms threatening the most unfavourable issue. This uncertainty of termination serves to shew how much the event is to be ascribed to the continued operation of the irritant—to the continued sense of injury (if it may be so expressed) which the constitution feels in one case more than in another.

Mr. Travers remarks that he has, again and again, left the bedsides of patients brought into the hospital pulseless and apparently moribund, without any apparent external injury, having suffered falls or blows so serious as to have induced the state of prostration to this alarming extent, and yet he has found them on the succeeding day, (to his great surprise) restored to the tone and tranquillity, comparatively speaking, of health. Reaction has, in these cases, been spontaneous, or nearly so, although gradual. Such patients have been supposed to labour under ruptures of the viscera or large blood-vessels—and the state of prostration to have been induced by the complication of the injury with internal hæmorrhagy :—and it has been only by the gradual return of healthy circulation, freedom of respiration, and corresponding sensations of relief, that this erroneous supposition has been corrected. "Now had such persons suffered topical injuries of a severe though reparable description, it is to my mind more than probable that re-action would have failed altogether ;—but had it, by favour of circumstances, been established, it is at least equally probable that it would have taken the form of excitement."

In other cases, days have elapsed before a perfect reaction, with relief, has been obtained, marking the most intense degree of shock, in the abstract, and shewing that complicated causes operate to render it destructive. In a large proportion of the graver accidents, the constitution recovers slowly from the first shock of the injury. The re-action is either deficient or in excess—as is additionally demonstrated by the unfavourable changes which take place in the condition of the parts injured.

"To conclude, the great mark of distinction between the cases of local injury which proceed steadily forward to convalescence, and those which place the life of the patient in peril, is the degree of implication of the nervous system. It is not well that this system should be unaffected, but the contrary. I have already adverted to that mode of irritation inseparable from any and every disease, which I consider to be neither more nor less than an extraordinary sympathy, rousing the powers of the system to a due resistance or exertion, and thus directly or indirectly promoting the process of recovery." 411.

Our next and concluding article will contain the following important subjects :—the theory of irritation—reciprocal relation of the vital functions—derangements of the nervous system, physical and functional—PATHOLOGY and TREATMENT of direct constitutional irritation—of the state of prostration, without re-action—of re-action—of constitutional irritation from injuries, &c. We shall then have laid before a wide range of the profession, one of the most extended analytical delineations which has ever been given of a medical work—a procedure which, we think, pays the author a higher compliment than can be conveyed in language however adulatory.

